# The Impact of Poor Motor Skills on Perceptual, Social and Cognitive Development: The Case of Developmental Coordination Disorder

**DOI:** 10.3389/fpsyg.2016.00311

**Published:** 2016-03-07

**Authors:** Hayley C. Leonard

**Affiliations:** School of Psychology, Faculty of Health and Medical Sciences, University of SurreySurrey, UK

**Keywords:** developmental coordination disorder, motor skill, embodiment, dynamic systems, physical activity, predictive control

Throughout development, we gain increasing control over our bodies, allowing us to move around our environment and manipulate and use objects. This developing motor control is key to our understanding of the properties of our environment (Piaget, 1953). Being able to crawl or walk affects infants' understanding of the distance between objects, as well as the feel of different surfaces and slopes (Adolph and Joh, 2007). They will also be exposed to different risks in the environment, leading to a change in the relationships with their caregivers as they learn to use social information, such as facial expressions or tone of voice, to guide their exploration (Campos et al., 2000). The development of motor skills can therefore be viewed as part of an interactive developmental process with perceptual, social, and cognitive abilities (Thelen, 1992), which is subject to the constraints of the body and the environment.

One theme of research which could help to provide more insight into these typical developmental processes is the study of neurodevelopmental disorders, in which expected interactions between different abilities may be disrupted. Motor difficulties have been highlighted in a number of neurodevelopmental disorders, including autism spectrum disorder (Provost et al., 2007), specific language impairment (Hill, 2001) and dyslexia (Nicolson et al., 2001), all of which have core deficits in other domains, such as language or social communication. The current article will focus on developmental coordination disorder (DCD), in which motor impairments are central to the diagnosis (see Table 1). DCD is relatively understudied and under-recognized in comparison to many other neurodevelopmental disorders, despite its prevalence of around 5–6% in the population (American Psychiatric Association, 2013). However, given the close connections between motor development and the other skills outlined above, this article will argue that DCD provides a model case for investigating the impact of poor motor skills on perceptual, social and cognitive development. A review of pertinent research will demonstrate the impact that poor motor skills can have on functioning in a number of different domains. Finally, the article will suggest that raising awareness of the relationships between these skills is a vital next step to aid earlier intervention for a range of perceptual, social and cognitive difficulties in individuals with motor difficulties. This will not only benefit those with DCD, but could also improve outcomes in a range of other neurodevelopmental disorders.

**Table 1:** **DSM-5 diagnostic criteria for developmental coordination disorder (American Psychiatric Association, 2013)**.

Criterion A	The acquisition and execution of coordinated motor skills is substantially below that expected given the individual's chronological age and opportunity for skill learning and use. Difficulties are manifested as clumsiness (e.g., dropping or bumping into objects) as well as slowness and inaccuracy of performance of motor skills (e.g., catching an object, using scissors or cutlery, handwriting, riding a bike, or participating in sports).
Criterion B	The motor skills deficit in Criterion A significantly and persistently interfere with activities of daily living appropriate to chronological age (e.g., self-care and self-maintenance) and impacts academic/school productivity, prevocational and vocational activities, leisure, and play.
Criterion C	Onset of symptoms is in the early developmental period.
Criterion D	The motor skills deficits are not better explained by intellectual disability (intellectual developmental disorder) or visual impairment and are not attributable to a neurological condition affecting movement (e.g., cerebral palsy, muscular dystrophy, degenerative disorder).

*DSM-5: Diagnostic and statistical manual of mental disorders (5th Edition)*.

## Action, perception, and cognition

The development of motor control has long been treated as the poor relation of “higher-order” skills such as perception and cognition (Rosenbaum, 2005). More recent theories of *embodiment*, however, stress the ongoing interactions between the brain, the body and the environment in every situation (e.g., Iverson and Thelen, 1999; Smith, 2005), demonstrating that perception and cognition guide movement and action as much as action influences perception and cognition (Adolph and Joh, 2007). Motor skills act as constraints on our actions, which affects our interactions with the environment and the way in which we learn about the correlations between our different senses and the information they provide (e.g., Smith, 2005). When motor control is affected, as it is in DCD, we can therefore expect to see differences in the way in which the environment is perceived and processed.

Studies of visuospatial and cognitive processes in DCD have reported wide-ranging difficulties and atypicalities. For example, individuals with DCD demonstrate poorer form and motion detection, as well as difficulties in a range of tasks assessing memory and cognitive control (see Wilson et al., 2013, for a meta-analysis). If perception, cognition, and motor control are viewed as functionally separate systems, it could be a challenge to explain these difficulties in a disorder diagnosed on the basis of motor impairments. In this case, it might be tempting to regard DCD as a more general learning disability, or to describe the perceptual and cognitive difficulties as reflections of co-occurrence of DCD with other neurodevelopmental disorders, such as attention deficit-hyperactivity disorder. If we focus on the integration of these symptoms, however, using an embodied approach, the relationships between motor, perceptual and cognitive impairments become clearer.

The close links between motor control and perception are demonstrated in the seemingly simple act of visually tracking a moving object. The act of smooth pursuit of an object improves over the first four to five months of life, and requires both control over posture (i.e., the ability to hold the weight of the head) and control over the eyes, as well as the coordination of eye and head movements (Von Hofsten, 2004). Smooth pursuit is intrinsically linked with object knowledge and the understanding of the physical properties of the environment. For example, when a moving object is momentarily hidden behind an occluder, infants must have a representation of the object and an understanding that it still exists in order to continue the smooth pursuit, and predictively look at the point from which the object will reappear (Adolph and Joh, 2007). The development of this ability relies on both experience with moving objects in the environment, and the rapid formation of new connections in the central nervous system, with brain areas of particular importance likely to be the parietal and prefrontal cortices, medial temporal areas of the visual cortex, and the cerebellum (Von Hofsten, 2004). Although brain imaging research is relatively limited within the DCD literature, these areas have all been implicated in the difficulties seen in DCD (Zwicker et al., 2009). Furthermore, although no research has been conducted with infants with DCD due to its identification during the school years, children of 7–12 years with a diagnosis demonstrate difficulties in smooth pursuit (Robert et al., 2014; Sumner et al., 2015). This suggests that early differences in brain development in these areas could constrain the typical improvement in smooth pursuit in infancy, which could have an impact on a range of perceptual and cognitive outcomes in DCD.

The ability to anticipate or predict the movement trajectory of an object is central not only to smooth pursuit, but also to the successful completion of actions toward objects (e.g., Wolpert, 1997). As an example, when an individual reaches for an object, it is proposed that the predictive system (or forward model) estimates the end point of the action, including the sensory information associated with that end point (such as the position of the hand in space, the feel of the object, etc.). This allows a comparison to be made during the action between the predicted and the actual sensory information being provided, and means that online, prospective changes can be made to ensure the success of the action, even when the environment is changing (Wolpert et al., 2001). If this predictive system were impaired, then individuals would have to rely on a slower feedback mechanism, which would be less effective in a dynamic, changing environment (Wilson, 2015). In DCD, it has been suggested that difficulties in motor planning (van Swieten et al., 2010; Pratt et al., 2014) and the online adaptation of movements (Hyde and Wilson, 2011; Ruddock et al., 2015) could reflect these underlying impairments in predictive control. Other issues with this system may also be evident in the problems with voluntary, effortful control over cognition (known as “executive functions”: Diamond, 2013), which are prevalent amongst individuals with DCD (see Leonard and Hill, 2015, for a review). As in smooth pursuit, both motor actions and executive functioning are subserved by underlying structural and functional connections between parietal and prefrontal cortices and the cerebellum (e.g., Diamond, 2000; Koziol et al., 2012; Ramnani, 2012). Thus, in DCD, atypical structure or functioning of these areas (e.g., Kashiwagi et al., 2009; Debrabant et al., 2013), reduced functional connectivity within this network (e.g., Zwicker et al., 2011), or an immature coupling between frontal and posterior control areas (Ruddock et al., 2015) could explain the difficulties seen in the disorder across a range of predictive control tasks. Further research into DCD provides an opportunity for investigating potential shared mechanisms underlying these forms of predictive control, as well as their neural bases, in both typical and atypical development.

## Motor skills, executive control, and social functioning

As outlined in the introduction to this article, developing motor control provides infants with opportunities for interacting with the social environment as well as their physical surroundings. As children grow older, motor skills are central to the types of play in which they engage, from the manual dexterity required for dressing up or making models out of toy bricks, to the physical play and team games seen in the playground. If the skills required for these activities are impaired, as in DCD, it is likely to have implications for social functioning and peer relations. Indeed, research into DCD investigating social activities, peer relations and social problems has reported significant correlations between motor abilities and parent-reported peer difficulties (Cummins et al., 2005; Green et al., 2006; Wagner et al., 2012). In playground observations, children with DCD were more likely to be alone and to be onlookers, rather than the center of a social group (Smyth and Anderson, 2000), and the relationship between motor coordination and self-reported loneliness by children with DCD was mediated by their participation in team sports (Poulsen et al., 2007). Poor motor development, and consequent low self-concept, could explain this reduced participation in motor activities and physical play in those with DCD (e.g., Wrotniak et al., 2006), which may result in social exclusion.

It is also possible that the close connections between brain areas related to motor and executive control could affect social functioning. Research in typically-developing children has reported that executive functions, such as cognitive flexibility, are highly correlated with social understanding and performance on theory of mind tasks in early and middle childhood (e.g., Hughes and Ensor, 2007; Bock et al., 2015). Further support for this view arises from research with children with attention deficit-hyperactivity disorder (ADHD), which is characterized by both executive functioning and social difficulties (Wehmeier et al., 2010). It is suggested that problems with flexibility could interfere with typical social interaction in those with ADHD, while difficulties inhibiting unwanted responses and regulating behavior could lead to peer rejection (e.g., Wheeler Maedgen and Carlson, 2000; Marton et al., 2009). As ADHD often co-occurs with DCD (American Psychiatric Association, 2013), and given that children with DCD often present problems in inhibition and self-regulation (e.g., Rahimi-Golkhandan et al., 2014; Leonard et al., 2015), it could be that social difficulties in DCD are related to these issues in executive control. Poor motor development could thus affect social functioning through at least two routes: first by limiting the potential to participate in physical activities and play, and second by affecting executive control and social understanding. As low motor competence is not only associated with DCD, but to a range of other neurodevelopmental disorders and environmental factors, it is of great importance that the identification of motor difficulties is improved, allowing appropriate early intervention in order to reduce the potential adverse effects on perceptual, cognitive and social development.

## Moving forward: An embodied approach

From the research outlined throughout this article, it is clear that motor development not only provides opportunities for the development of a range of perceptual, social, and cognitive skills, but is influenced in turn by these abilities in an interactive process. Using an embodied approach, it is possible to consider the relationships and constant interactions between domains as part of a dynamic system with shared underlying mechanisms, such as predictive control. Improving our understanding of these shared mechanisms will be important for developing more integrated interventions for those with a range of difficulties. Furthermore, raising awareness of the impact of poor motor skills on perception, cognition and social development amongst psychologists, teachers, parents, and practitioners will be vital in improving outcomes for those with motor difficulties. DCD provides a model case for further investigation, due to the known motor impairments in the disorder. However, research into this area will have implications for a wide range of individuals with neurodevelopmental disorders, as well as for those with low motor competence associated with a number of different factors.

## Author contributions

The paper was conceived, drafted, and revised by HL as the sole author.

### Conflict of interest statement

The author declares that the research was conducted in the absence of any commercial or financial relationships that could be construed as a potential conflict of interest.
